# Massive Transfusion Protocol Predictive Modeling in the Modern Electronic Medical Record

**DOI:** 10.1097/AS9.0000000000000109

**Published:** 2021-12-14

**Authors:** William Shihao Lao, Jessica L. Poisson, Cory J. Vatsaas, Christopher J. Dente, Allan D. Kirk, Suresh K. Agarwal, Steven N. Vaslef

**Affiliations:** From the * Department of Surgery, Duke University Medical Center, Durham, NC; † Department of Pathology, Duke University Medical Center, Durham, NC; ‡ Department of Surgery, Emory University School of Medicine, Atlanta, GA; § Surgical Critical Care Initiative (SC2i), Bethesda, MD.

## Abstract

**Objectives:**

Integrate a predictive model for massive transfusion protocol (MTP) activation and delivery in the electronic medical record (EMR) using prospectively gathered data; externally validate the model and assess the accuracy and precision of the model over time.

**Background:**

The Emory model for predicting MTP using only four input variables was chosen to be integrated into our hospital’s EMR to provide a real time clinical decision support tool. The continuous variable output allows for periodic re-calibration of the model to optimize sensitivity and specificity.

**Methods:**

Prospectively collected data from level 1 and 2 trauma activations were used to input heart rate, systolic blood pressure, base excess (BE) and mechanism of injury into the EMR-integrated model for predicting MTP activation and delivery. MTP delivery was defined as: 6 units of packed red blood cells/6 hours (MTP1) or 10 units in 24 hours (MTP2). The probability of MTP was reported in the EMR. ROC and PR curves were constructed at 6, 12, and 20 months to assess the adequacy of the model.

**Results:**

Data from 1162 patients were included. Areas under ROC for MTP activation, MTP1 and MTP2 delivery at 6, 12, and 20 months were 0.800, 0.821, and 0.831; 0.796, 0.861, and 0.879; and 0.809, 0.875, and 0.905 (all *P* < 0.001). The areas under the PR curves also improved, reaching values at 20 months of 0.371, 0.339, and 0.355 for MTP activation, MTP1 delivery, and MTP2 delivery.

**Conclusions:**

A predictive model for MTP activation and delivery was integrated into our EMR using prospectively collected data to externally validate the model. The model’s performance improved over time. The ability to choose the cut-points of the ROC and PR curves due to the continuous variable output of probability of MTP allows one to optimize sensitivity or specificity.

## Abstract 2

Mini-Abstract: Prospectively gathered data were used to integrate a predictive model for massive transfusion activation and delivery into the EMR. Receiver operating characteristic (ROC) and Precision-recall (PR) curves at 6, 12, and 20 months were used to show the adequacy of the model and its improvement over time.

## INTRODUCTION

Management of the modern polytrauma patient is an exercise in carefully controlled chaos wherein the astute clinician must rapidly intake and process an avalanche of data while simultaneously making time-sensitive and life-saving decisions. The activation and use of an institution’s massive transfusion protocol (MTP) is one such decision that the clinician will face. These protocols are a key component of modern resuscitation strategies, which were first developed by the military and have gained widespread acceptance in civilian trauma centers.^1^

There exists significant evidence that early transfusion of blood products as part of a damage control resuscitation strategy improves patient outcomes by avoiding the dreaded lethal triad of acidosis, coagulopathy, and hypothermia.^2,3^ Delays in administering blood products to the 3% to 5% of civilian trauma patients presenting with hemorrhagic shock and trauma-related coagulopathy is associated with significantly increased morbidity and mortality.^4^ However, overuse of this resource-intensive protocol can severely deplete an institution’s limited resources.^5^ Furthermore, over-transfusion of patients who do not require large numbers of blood products exposes them to significant risks of transfusion-related complications such as volume overload, acid-base derangements, electrolyte abnormalities, and transfusion-associated acute lung injury.^6,7^

Current standard of practice typically dictates that the decision to activate a MTP is left to the discretion of the trauma team leader and is often based on nothing more than clinical gestalt. An inherent weakness associated with clinical judgment is the fact that it is, as demonstrated by Pommerening et al, often marginally accurate at best.^8^ To counteract this deficiency, multiple MTP prediction models have been developed.^9^ Unfortunately, many of these models possess significant drawbacks. For example, the trauma-associated severe hemorrhage (TASH) score developed by Yücel et al in 2006 prioritizes accuracy of prediction and is dependent on the input of up to 8 variables.^10^ The resulting model is reliable and accurate yet can be somewhat cumbersome to use at the bedside.^11^ On the other hand, the Assessment of Blood Consumption (ABC) score developed by Cotton et al in 2009 prioritizes ease-of-use by using 4 dichotomized variables.^12^ The ABC model, while practical and accurate, provides only a yes/no output without room for nuance.^13^ The Emory model, developed at Emory University by Mina et al,^14^ is attractive because of its simplicity of use, input of only 4 variables, output of probability of MTP as a continuous variable, prior external validation of the model using a multicenter dataset,^15^ and, more recently, an internal prospective validation.^16^

One significant barrier to the routine use of MTP predictive modeling is the fact that they all require a dedicated person performing the calculation, be it via a web-based tool or a smartphone application. With the implementation of electronic medical record (EMR) systems came an opportunity for creating integrated predictive models to serve as real-time clinical decision support tools (CDSTs). CDSTs have the potential to serve as both reminders and methods of cognitive offloading, effectively adding an added layer of safety in patient care.^17–19^

With this in mind, we sought to integrate the Emory MTP prediction model into our institution’s EMR; develop a real-time best practice advisory (BPA) notification in the EMR for the need of MTP activation and delivery; and to permanently embed the output probability of MTP into the EMR trauma flowsheet. Furthermore, using prospectively collected MTP probability data, we sought to externally validate and assess the accuracy and precision of the predictive model. We hypothesized that the model’s accuracy and precision would improve over time as more data were accrued and the appropriateness of activating MTP was regularly scrutinized.

## METHODS

### Data Collection

Upon obtaining institutional review board approval, data were gathered over a 20-month period between April 2018 and November 2019. All level 1 and 2 trauma activations for patients aged 15 and older at Duke University Hospital in Durham, North Carolina, were extracted from the institution’s trauma registry. Level 3 activations comprising less seriously injured patients were excluded. MTP activation was tracked through the institution’s blood bank database. MTP delivery was defined using one of two common definitions: 6 units of packed red blood cells (pRBCs) over 6 hours (labeled as MTP1 delivery) or 10 units of pRBCs over 24 hours (labeled as MTP2 delivery). The actual rate of transfusion was determined by chart review.

### Model Description

The Emory MTP prediction model has been previously described.^14^ Briefly, it utilizes four input variables: initial heart rate (HR), systolic blood pressure (SBP), base excess/deficit, and mechanism of injury (blunt, stab wound, or gunshot wound). A type of regression known as LASSO (least absolute shrinkage and selection operator), which optimizes bias-variance trade-off and produces a less complex model that may be more predictive and interpretable, is used to calculate model coefficients. The output is probability of MTP, which is delivered as an on-screen pop-up, or BPA, in real time. When an automatic BPA was not issued due to missing value, the MTP probability was hand calculated using the same model from variables extracted by chart review.

### Model Integration Into the EMR

Duke University Health System utilizes Epic Hyperspace as its EMR (Epic Systems Corporation, Verona, WI). During a trauma resuscitation, a nurse scribe inputs three of the four variables needed to calculate the probability of MTP (systolic blood pressure, heart rate, and mechanism of injury—gun shot wound, stab wound, or blunt trauma). The fourth variable, base deficit, is extracted from the first blood gas performed in the emergency department. Once all of these variables are incorporated into the EMR, Epic identifies level 1 and level 2 trauma activations and calculates the probability of MTP, according to code written into the Epic program. The output appears in the EMR as an on-screen best practice advisory pop-up. The probability of MTP is also embedded permanently into the electronic trauma flowsheet within the EMR.

### Statistical Analyses

Receiver operating characteristic (ROC) and precision-recall (PR) curves were constructed at 6, 12, and 20 months to assess the adequacy of the model for predicting MTP activation, MTP1 delivery, and MTP2 delivery and to determine if the model improved over time as more data were accrued (MedCalc Software Ltd, Ostend Belgium and Microsoft Excel, Microsoft Corporation, Redmond, WA). PR curves were used in conjunction with ROC curves due to the imbalanced dataset, that is, a dataset in which the incidence of true negatives, in this case, far exceeds that of true positives^20^.

## RESULTS

### Patient Demographics

Over the course of the study period, a total of 1412 Level 1 and 2 trauma activations meeting our inclusion criteria were registered. Of these, 250 were excluded due to missing data (249 due to lack of a base deficit and 1 due to a lack of SBP in a patient with a left ventricular assist device). Of the remaining 1162 patients included in the analysis, 931 had a BPA issued and 231 had their MTP probability hand calculated based on the first available base deficit value obtained from a blood gas.

Table 1 displays demographics of the entire patient population. Of note, the mean age was 43.9 years and there was a male preponderance (73.3%). Penetrating traumas accounted for 30.3% of total mechanisms of injury. Mean injury severity score was 12.6. The mean SBP was 127.9 mm Hg and mean HR was 91.4 BPM. The mean base excess was –3.0. Mean hospital length of stay was 9.4 days overall and 3.5 days in the ICU. Overall in-hospital mortality was 9.6% with 3.9% of patients dying within 24 hours of presentation.

**TABLE 1.** Patient Characteristics (n = 1162)

Age, Mean (SD), years	43.9 (20.4)
Males, n (%)	852 (73.3)
Penetrating trauma, n (%)	352 (30.3)
Systolic blood pressure, mean (SD), mm Hg	127.9 (32.8)
Heart rate, mean (SD), beats/min	91.4 (23.6)
Base excess, mean (SD), meq/L	–3.0 (5.3)
Injury Severity Score, mean (SD)	12.6 (11.0)
ICU length of stay, mean (SD), days	3.5 (7.5)
Hospital length of stay, mean (SD), days	9.4 (15.3)
24-hour mortality, n (%)	44 (3.8)
In-hospital mortality, n (%)	101 (8.7)

SI conversion factor: To convert Base excess to mmol/L, multiply value by 1.

Table 2 displays the demographics of the patients in each of the defined groups: MTP activation, MTP1 delivery, and MTP2 delivery. The groups are not mutually exclusive, so that, for example, patients that met the definition of both MTP1 delivery and MTP2 delivery are included in both groups.

**TABLE 2.** Group Characteristics Using the Various Definitions of Massive Transfusion

	MTP Activation	MTP1 Delivery	MTP2 Delivery
n	88	73	53
Age, mean (SD)	37.0 (17.5)	37.8 (16.9)	38.3 (16.4)
Males, n (%)	69 (78.4)	60 (82.2)	44 (83.0)
Mechanism of injury			
Gunshot wound, n (%)	36 (40.9)	28 (38.4)	21 (39.6)
Stab wound, n (%)	8 (9.1)	3 (4.1)	3 (5.7)
Blunt, n (%)	44 (50.0)	42 (57.5)	29 (54.7)
Systolic blood pressure, mean (SD), mm Hg	91.8 (47.2)	87.9 (41.5)	83.7 (43.4)
Heart rate, mean (SD), beats/min	90.9 (41.7)	93.1 (40.5)	93.7 (43.2)
Base excess, mean (SD), meq/L	-9.5 (7.2)	-10.5 (6.8)	-12.2 (6.9)
Injury Severity Score, mean (SD)	23.6 (11.4)	26.2 (11.8)	25.4 (12.8)
ICU length of stay, mean (SD), days	6.5 (6.5)	8.2 (9.3)	7.8 (9.6)
Hospital length of stay, mean (SD), days	15.8 (18.4)	19.9 (20.8)	18.5 (19.8)
Units PRBC given in first 24 hours, mean (SD)	15.4 (16.7)	19.3 (16.2)	23.8 (17.0)
24-hour mortality, n (%)	19 (21.6)	16 (21.9)	13 (24.5)
In-hospital mortality, n (%)	31 (35.2)	26 (35.6)	21 (39.6)

SI conversion factor: To convert Base excess to mmol/L, multiply value by 1.

MTP Activation (massive transfusion protocol activated), MTP1 delivery (6 units PRBCs in 6 hours), MTP2 delivery (10 units PRBCs in 24 hours).

### Model Performance and Validation

**FIGURE 1.**
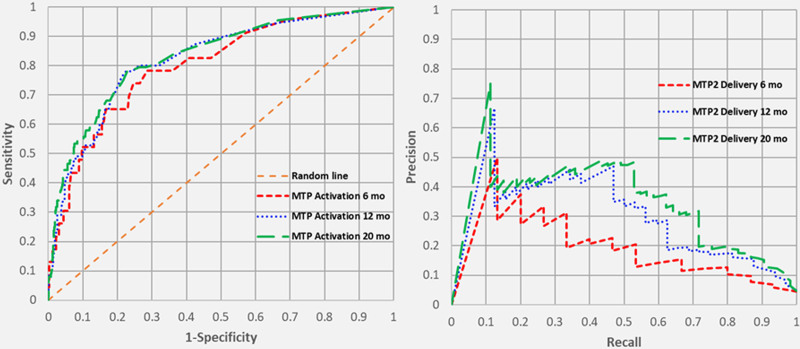
Receiver operating characteristic and precision-recall curves for MTP activation over time.

**FIGURE 2.**
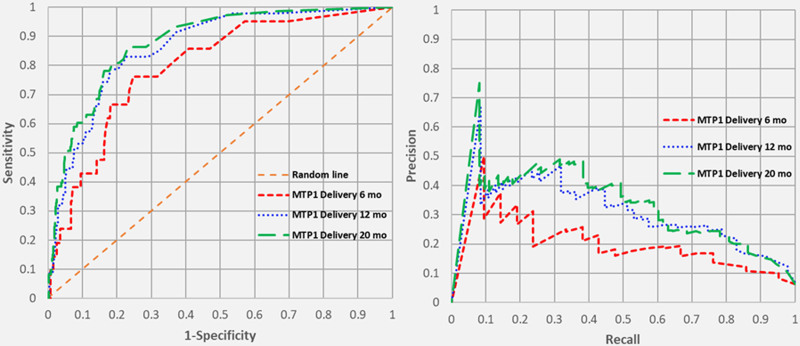
Receiver operating characteristic and precision-recall Curves for MTP1 delivery over time.

**FIGURE 3.**
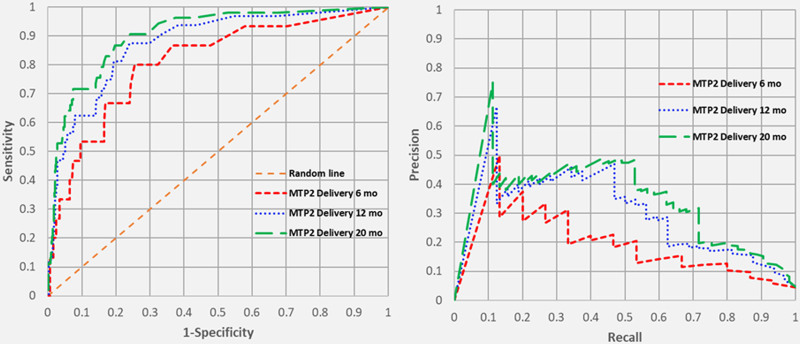
Receiver operating characteristic and Precision-Recall Curves for MTP2 delivery over time.

All ROC and PR curves for MTP activation, MTP1 delivery, and MTP2 delivery yielded moderate to strong predictive ability which improved over time. Figure 1 shows the ROC and PR curves for MTP activation. The areas under the ROC curves for MTP activation at 6, 12, and 20 months were 0.800, 0.821, and 0.831 (all *P* < 0.001), while the areas under the PR curves for the same time periods were 0.304, 0.353, and 0.371 (all *P* < 0.05 compared with the no skill lines). Figure 2 shows the ROC and PR curves for MTP1 delivery at 6, 12, and 20 months. The areas under the ROC curves were 0.796, 0.861, and 0.879 (all *P* < 0.001), respectively, and the areas under the PR curves for these time periods were 0.201, 0.313, and 0.339 (all *P* < 0.05). Finally, Figure 3 shows the ROC and PR curves for MTP2 delivery. The areas under the ROC curves at 6, 12, and 20 months were 0.809, 0.875, and 0.905 (all *P* < 0.001), while the areas under the PR curves were 0.194, 0.305, and 0.355 (all *P* < 0.05).

## DISCUSSION

The ability to rapidly interpret a large volume of clinical data and predict the necessity of massive transfusion in a trauma patient is an important skill in the arsenal of any physician engaged in acute resuscitation. Our EMR-integrated model, built upon an existing model developed and validated at Emory, demonstrates the feasibility of using our existing medical record system to assist in clinical decision making without being cumbersome or intrusive. It also demonstrates the generalizability of a CDST established in one health system and integrated into another, a critical characteristic of algorithmic decision support. Both Emory and Duke are partners in the Surgical Critical Care Initiative (SC2i), which is funded by the Department of Defense’s Defense Health Program and was established in 2013 to develop CDSTs to help manage complex and critically injured patients. Our work was a deliberate attempt to export a CDST developed at one site and integrate it into our health system’s EMR. This clinical decision support tool assists the physician, in real time, in deciding whether or not to activate MTP. This is an important consideration due to the intense blood bank and nursing resources associated with MTP, as well as the cost and risks of administering multiple blood products.

There are several advantages to having this model integrated into the EMR. First, it eliminates the need to have the application on a smartphone, as is currently done at Emory. This avoids any potential HIPAA compliance issues with having patient data stored on personal devices and also reduces time and manpower by not having to manually input variables redundantly into a personal device. The nurse scribe inputs the variables into the EMR trauma flowsheet for every trauma patient, regardless of whether or not the model is being used. Second, the BPA is embedded in the EMR, so it is permanent and trackable. Third, as automation of data entry into the EMR matures, artificial intelligence (AI) can be used to optimize the model’s sensitivity and specificity.

The ROC curves demonstrate that our model has a moderate-to-strong predictive ability which increases with time and data accrual. The reason the Emory model by Mina and colleagues was chosen is that it inputs 3 out of 4 continuous variables and creates an output that is also a continuous variable. A major strength of the model, which differentiates it from other MTP prediction models, is the continuous variable output, which allows one to choose a cutoff probability that optimizes sensitivity or specificity. Hence, the ROC curve can be periodically “re-calibrated” to change the cutoff point depending on how the clinician wants to balance the “false alarms” (false positives) and the false negatives. Arguably, most clinicians would want to minimize the latter, thereby maximizing the sensitivity. However, in a very resource-limited environment, one might opt to limit the number of false positives to increase the specificity of the model.

Our results provide further evidence of external validation of the Emory model. As originally described by Mina et al, the Emory model used single-institution data to construct and validate the model to predict MTP activation.^14^ Their AUC for the ROC curve was 0.96, which is higher than the AUC of 0.831 utilizing our data to predict MTP activation. A plausible explanation for their higher AUC is that they included all trauma admissions, regardless of mechanism of injury or injury severity. We included only patients who were triaged with the two highest levels of trauma activation. Therefore, the Emory dataset included more “true negatives,” as evidenced by their prevalence of MTP activation of 3.6%, compared to our prevalence of 7.6%. Hodgman et al utilized a database from the Prospective, Observational, Multicenter, Major Trauma Transfusion (PROMMTT) study to retrospectively validate the Emory model.^15^ The PROMMTT data resulted in AUCs for the ROC curves ranging from 0.694 to 0.711, depending on the definition of massive transfusion used. Our data, in comparison, yielded AUCs of 0.879 for MTP1 delivery (6 units PRBCs in 6 hours) and 0.905 for MTP2 delivery (10 units PRBCs in 24 hours). One explanation for the higher AUCs using our data are that the patients in the PROMMTT database were more severely injured, on average, than our patients. The prevalence of massive transfusion administration using our definition of MTP1 delivery was 31.7% for the PROMMTT data compared with a prevalence of 6.3% in our dataset. For MTP2 delivery, the prevalence was 23.9% for the PROMMTT data versus 4.6% for our data. Thus, we had more true negatives than in the PROMMTT data, resulting in ROC curves that appear to better predict massive transfusion activation or delivery. Another possible explanation for the higher AUCs in our data compared with the PROMMTT data are that our data comes from a single institution, so there may be less trauma surgeon variability as to when to activate MTP. The PROMMTT study included 10 trauma centers, so it is plausible that there was more variable practice regarding activation of MTP.

We also wanted to call attention to the use of PR curves, in conjunction with ROC curves, when interpreting imbalanced datasets. ROC curves have a tendency to paint an overly optimistic picture in such cases, where the number of true positives (or prevalence of the outcome of interest) is low. In our database, actual MTP activation occurred in only 88 (7.6%) of the traumas, MTP1 delivery occurred in 73 (6.3%), and MTP2 delivery occurred in 53 (4.6%). PR curves have the ability to demonstrate the relationship between precision (= positive predictive value) and recall (= sensitivity) for every possible cutoff while ignoring true negative rates, which are irrelevant. Whereas the ROC curve is compared with the “no skill,” or random line depicted as X = Y, the no skill line for the PR curve is the prevalence of that particular outcome. The PR curves for MTP activation, MTP1 delivery, and MTP2 delivery were all significantly better than the no skill lines in this dataset.

As we continue to prospectively accrue more data, our model’s precision and accuracy may continue to improve. Periodic review, scrutiny, and feedback of the appropriateness of trauma surgeon MTP activation and MTP delivery within our institution may decrease variability of practice and the resulting number of false negatives and false positives.

Currently, there are several limitations to this model. First, the rate-limiting step to output the probability of MTP in real time is obtaining the blood gas result so that the base deficit can be inputted into the model. At our institution, the turnaround time to receive a blood gas result is between 10 and 15 minutes. Potentially, point-of-care testing in the Emergency Department (ED) could decrease the turnaround time. Second, the computer code to calculate the probability of MTP in the EMR, as originally written, results in a pop-up best practice advisory only if the patient is still physically in the ED. If the patient has already been transported to the operating room, for example, before the blood gas result returns, the BPA is not issued. This is the reason that 231 of the 1162 patients in our dataset had the probability of MTP hand-calculated based on the first available blood gas result, which most commonly occurred in the operating room or the intensive care unit. Third, individual chart review had to be undertaken to determine the actual rates of transfusion of PRBCs to see if the rates met our definitions of MTP1 delivery or MTP2 delivery. Revisions to the computer code to address these last two limitations are under investigation to better automate the model. Finally, the variability in the model’s performance, as alluded to above, should be viewed as a limitation at this time. The model may perform better in some trauma centers than others, depending on the level of trauma severity a center sees, as well as the variability of activating MTP from surgeon to surgeon. More consistent practice within and between trauma centers might mitigate this last limitation going forward.

It is important to emphasize that this prediction model is a clinical decision support tool that must be used in conjunction with best clinical judgment. A patient presenting with shock from nonhemorrhagic causes may result in a high probability of MTP advisory because of the way the model heavily weighs the base deficit. For example, a trauma patient that comes in after asphyxiation or drowning may have a large base deficit without evidence of hemorrhage and may flag a high probability of MTP. The trauma surgeon, in such cases, should rely on the clinical context and not activate MTP. As it currently stands, then, MTP activation remains a clinician-triggered decision. As predictive models continue to improve, one dilemma future physicians may have to face is whether to accept clinical decisions made by AI. In other words, when, if ever, will a model be considered accurate enough to allow for automatic triggering of MTP activation?

## CONCLUSIONS

Using only four simple and commonly gathered variables prospectively collected over a 20 month period, we were able to create a flexible and EMR-integrated clinical prediction model for the activation and use of MTP in trauma resuscitations. The inherent strength of this model is apparent as the area under ROC and performance steadily increases over time as more data are accrued, allowing us to periodically recalibrate the curves in order to balance the sensitivity and specificity of the model. In the future, we will be studying whether the use of such a model has any impact on MTP use and patient outcomes.

## ACKNOWLEDGMENTS

Shelley Gillman and Michael Noltner contributed to the EMR integration of the MTP prediction model.
